# The Impact of Aging on the Lung Alveolar Environment, Predetermining Susceptibility to Respiratory Infections

**DOI:** 10.3389/fragi.2022.818700

**Published:** 2022-01-19

**Authors:** Jordi B. Torrelles, Blanca I. Restrepo, Yidong Bai, Corinna Ross, Larry S. Schlesinger, Joanne Turner

**Affiliations:** ^1^ Population Health and Host-Pathogen Interactions Programs, Texas Biomedical Research Institute, San Antonio, TX, United States; ^2^ School of Public Health in Brownsville, University of Texas Health Houston, Brownsville, TX, United States; ^3^ South Texas Diabetes and Obesity Institute, University of Texas Rio Grande Valley, Edinburg, TX, United States; ^4^ Department of Cell Systems and Anatomy, UT-Health San Antonio, San Antonio, TX, United States; ^5^ Soutwest National Primate Research Center, Texas Biomedical Research Institute, San Antonio, TX, United States

**Keywords:** aging, lung environment, oxidative stress, respiratory infections, tuberculosis

## Abstract

Respiratory infections are one of the top causes of death in the elderly population, displaying susceptibility factors with increasing age that are potentially amenable to interventions. We posit that with increasing age there are predictable tissue-specific changes that prevent the immune system from working effectively in the lung. This mini-review highlights recent evidence for altered local tissue environment factors as we age focusing on increased tissue oxidative stress with associated immune cell changes, likely driven by the byproducts of age-associated inflammatory disease. Potential intervention points are presented.

## General Introduction

The elderly population will double to 2 billion by 2050 (Publications, 2019). Age-associated cellular decline begins to be notable in our 40s and accelerates in our 60s (Jones et al., 2002; Massudi et al., 2012; López-Otín et al., 2013; Schneider et al., 2021). While aging represents a complex progression involving many physiological and physical changes in metabolism and endurance, the field of aging is advancing steadily, with greater understanding of the changes in cellular processes, and the critical role that tissue-specific health plays in how we age. However, the molecular events driving age-dependent physiological and physical decline and the molecular thresholds at the tissue and cellular levels that determine the point(s) of no return or so-called *tipping point(s)*, resulting in non-recoverable tissue or systemic dysfunction are largely unknown. Here, we will highlight how and when some aspects of age-associated cellular decline occur in the pulmonary space, a critical organ that interfaces directly with the environment and is prone to infections. Such information can help develop therapeutic strategies to enhance healthy pulmonary aging in a timely manner to mitigate or delay deleterious events.

## The Lung Alveolar Environment During Aging

**FIGURE 1:**
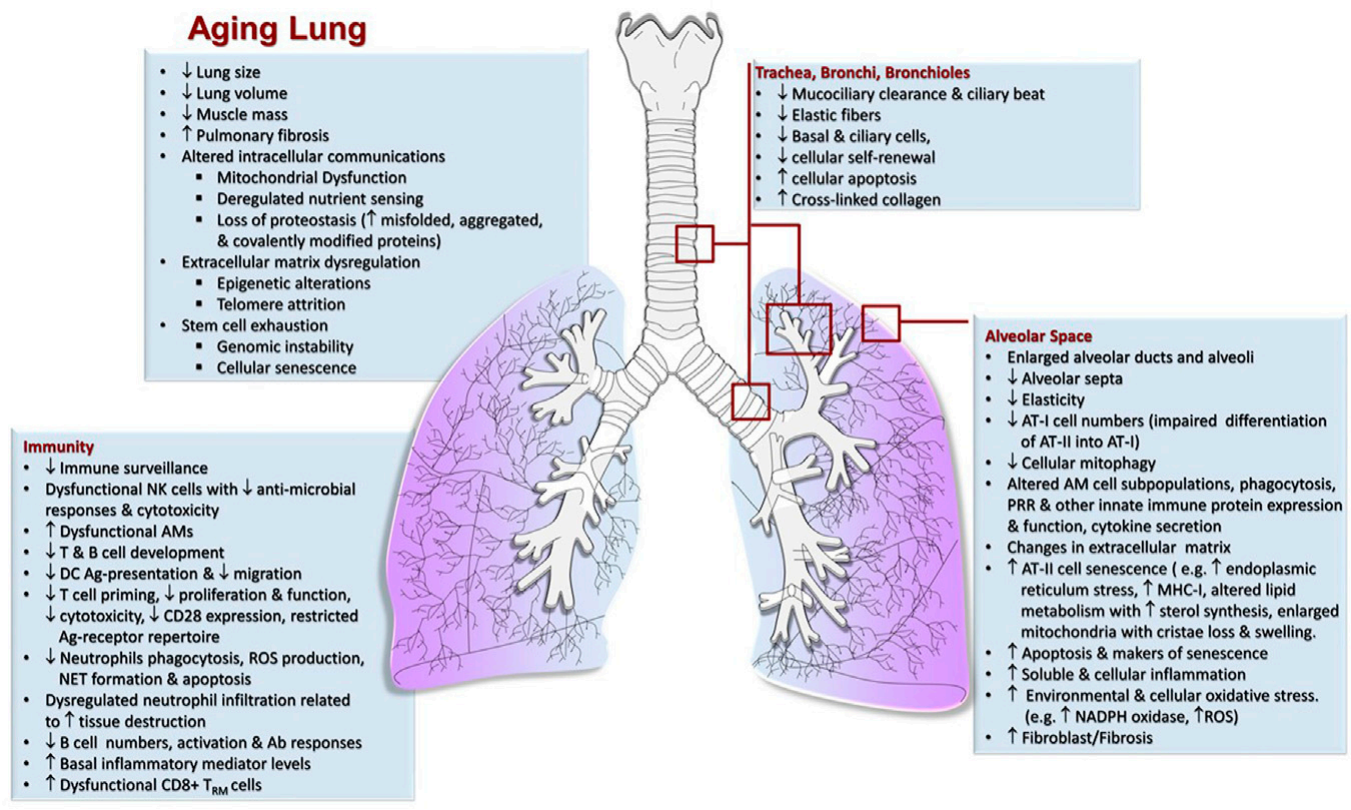
Characteristics of the Aging lung. Mechanical, physiological and immunological (increased inflammation/immunosenescence) changes in the lung that take place in elderly individuals increasing the risk of airway clearance failure and susceptibility to respiratory infections.

**FIGURE 2:**
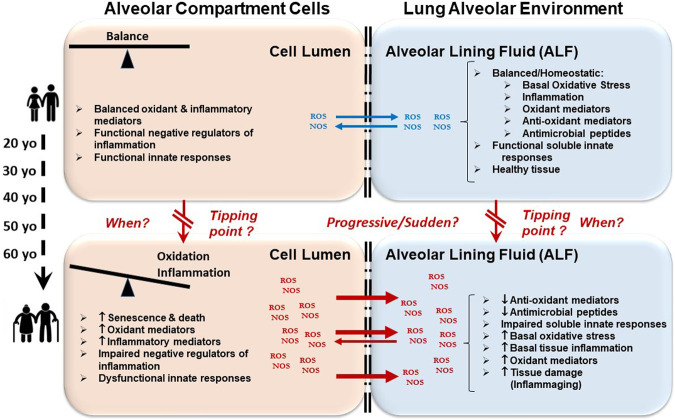
Oxidative and inflammatory environment in the lung leads to immunological impartment and susceptibility to respiratory infections. As we age, cellular senescence occurs. There are also alterations in cellular metabolic processes involving energy production and consumption among others. These events lead to an accumulation of oxidative stressors (ROS/NOS) in the lumen of alveolar compartment cells (left boxes) causing increased cellular inflammation that cannot be efficiently regulated. Subsequently, this accumulation of oxidative stressors outflows into the alveolar environment (e.g., upon cell death) (right boxes). This outflow causes lung tissue inflammation and damage, and also drives the oxidation and impairment of lung soluble and cellular immunomodulators. Altogether, these events contribute to the elderly becoming susceptible to acute and chronic respiratory infections and diseases.

As we age, there are changes in the physical environment and mechanical function of the lung that influence breathing and contribute to the increased susceptibility of the elderly to many infections (Schneider et al., 2021; Dyer, 2012; Vaz Fragoso and Lee, 2012; Haynes, 2020). Aging-related changes in the respiratory system generally include structural changes in the thoracic cage and lung parenchyma, abnormal lung function including ventilation and gas exchange abnormalities, decreased exercise capacity, airway nerve impairment, and reduced respiratory muscle strength and elasticity; each of which affect the cough reflex, sneezing or breathing (Figure 1) (Schneider et al., 2021). These progressive changes cause a decline in lung function and impaired immunological responses (Janssens et al., 1999; Lowery et al., 2013; Sharma and Goodwin, 2006; van Oostrom et al., 2018; Chalise, 2019; Thomas et al., 2019; Cho and Stout-Delgado, 2020; Haynes, 2020). To avoid cumulative damage, lung-resident cells rely on a robust homeostatic balance of stress response and associated inflammatory pathways; however, there is likely a tipping point (Figure 2), where age-associated changes finally overwhelm these control mechanisms leading to an increasing oxidative environment and irreversible damage (Moliva et al., 2014; Schöttker et al., 2015; Schneider et al., 2021). These factors combined with reduced lung-specific homeostatic immune activities facilitate entry of pathogens into the upper and lower respiratory tract leading to an increased propensity for infection (Vaz Fragoso and Lee, 2012; Haynes, 2020).

### The Lung Mucosa

The alveolus is lined with lung mucosa, composed of a surfactant lipid layer and an aqueous-hypophase called alveolar lining fluid (ALF) (Torrelles and Schlesinger, 2017). ALF contains innate soluble components with the primary role of maintaining proper pulmonary function (Notter, 2000; Fronius et al., 2012; Torrelles and Schlesinger, 2017). Our studies and others indicate that lung tissue in the elderly (in humans and mice) has a high basal inflammation and oxidative stress that leads to dysfunction of critical innate soluble and cellular components that could drive host susceptibility to respiratory infections and diseases [e.g., influenza, pneumonia, tuberculosis (TB)] (Toapanta and Ross, 2009; Shivshankar et al., 2011; Canan et al., 2014; Moliva et al., 2014; Piergallini and Turner, 2018a; Keilich et al., 2019; Bulut et al., 2020; Harpur et al., 2021). Thus, defining when and how these changes occur at the cellular and molecular level is critical to understand age-associated lung-specific pathologies and aging in general. Published studies demonstrate that the elderly have basal inflammation (e.g., elevated levels of TNF-α, IL-6, IL-1β, and IL-12), as well as increased protein oxidation with elevated levels of proteins with carbonyl and nitrotyrosine residues indicative of oxidation by reactive oxygen (ROS) and nitrogen (RNS) species, respectively, and reduced protein function in the lung (Moliva et al., 2014; Moliva et al., 2019). Indeed, studies focused on studying the lung mucosa composition in the elderly showed increased levels of innate surfactant proteins and components of the complement system, but with diminished function (Moliva et al., 2014). Dysfunction of innate soluble components of the lung mucosa is linked directly to high levels of oxidative stress [(e.g., elevated levels of ROS/RNS and myeloperoxidase (MPO)], which impair their function, and oxidizes critical surfactant lipids involved in keeping the lung alveolar space integrity and functionality (Moliva et al., 2014).

Studies addressing the impact of the human lung mucosa from the elderly on microbial pathogenesis are very limited. Upon reaching the alveolar space, respiratory pathogens come in close contact with the lung mucosa before and after their encounter with host alveolar compartment cells [e.g., type 1 and 2 epithelial cells (ATs) and alveolar macrophages (AMs)]. Our previous studies show that homeostatic innate soluble components of the ALF can quickly alter the cell envelope surface of pathogens upon contact, influencing subsequent pathogen*-*host cell interactions and infection outcomes (Arcos et al., 2011; Arcos et al., 2015; Arcos et al., 2017; Hill et al., 2017; Scordo et al., 2017; Scordo et al., 2019; Haynes, 2020). Alterations of a pathogen cell envelope is mainly due to homeostatic hydrolytic activities (hydrolases), primarily responsible for maintaining lung health, that modify pathogens and influence their subsequent interactions with host cells (Arcos et al., 2011; Hill et al., 2017; Torrelles and Schlesinger, 2017). In this context we have demonstrated that ALF from healthy 60 + year old individuals is dysfunctional, with loss of homeostatic hydrolytic capacity and impaired innate soluble responses linked to high local oxidative stress, compared to healthy 18- to 45-year-old individuals (Moliva et al., 2014). Importantly, a pathogen such as *Mycobacterium tuberculosis* exposed to ALF from the elderly demonstrates increased virulence *in vitro* and *in vivo* indicating that impaired innate responses in the lung may play a critical role in susceptibility to respiratory infection as we age (Moliva et al., 2019). In this context, two major cell populations in the alveolar space are AMs and ATs. Our studies indicate that after exposure to ALF from the elderly, *M. tuberculosis* doubles its intracellular growth rate in AMs, further indicating the impact of ALF dysfunction as we age (Moliva et al., 2019). Thus, efforts to mitigate oxidative stress in the lungs prior to a so-called *tipping point(s)* may reduce inflammation and aid in functional recovery of important innate immune-modulators against respiratory pathogens (Piergallini and Turner, 2018b; Iddir et al., 2020; Michaeloudes et al., 2020).

Evidence for a higher oxidative environment of the elderly lung is also supported by proteomics studies in other tissues and *in-vitro* studies with purified mitochondria from old mice (Moliva et al., 2014; Stauch et al., 2015; Ingram and Chakrabarti, 2016; Gómez-Serrano et al., 2018; Moliva et al., 2019; Ubaida-Mohien et al., 2019; Yang et al., 2019). However, the mechanism behind elevated oxidative stress in the lungs as we age is still not completely understood. Based on studies in other tissues, the oxidative pulmonary environment may be attributed in part to mitochondrial dysfunction. Mitochondria are primarily responsible for producing ATP (Anderson et al., 2019) and are found in greater numbers in tissues and organs where energy needs are high (e.g., muscle, heart, brain, liver and kidney). However, few studies have focused on the lung despite its direct exposure to oxygen, large consumption of energy (Liu and Summer, 2019), and high risk of dysfunction and disease in older age as mitochondrial function declines (Short et al., 2005; Kim et al., 2015; Haas, 2019; Sekhar M.D. et al., 2019). Thus, interventions to address the management of mitochondria-induced oxidative stressors (e.g. reactive oxygen and nitrogen oxidative species, ROS, NOS) in the lung tissue should mitigate lung oxidative stress and inflammation, and lead to improved resistance of the elderly to respiratory infections.

### Alveolar Macrophages During Aging

Aging is associated with a weakening of immune function, called cellular immunosenescence. In addition to this process there is a baseline level of dysfunctional inflammation in old age termed inflammaging (Franceschi et al., 2000; Moliva et al., 2014; Frasca and Blomberg, 2016; Fulop et al., 2017; Lafuse et al., 2019; Moliva et al., 2019). The relationship between inflammaging and dysfunctional innate immunity is understood poorly, especially in the lung. These changes are thought to be critical to understanding why the elderly are at an increased risk of developing and succumbing to malignancies, autoimmunity (Linton and Dorshkind, 2004; Pawelec et al., 2010), and infectious diseases such as TB (Piergallini and Turner, 2018b; Lafuse et al., 2019; Moliva et al., 2019).

AMs live in a unique tissue environment and we are only now gaining insight into the local environmental factors that impact AM phenotype and function (Guth et al., 2009; Davies and Taylor, 2015; Allard et al., 2018; Papp et al., 2018; Hoeksema and Glass, 2019; Lafuse et al., 2019). AM responses enable us to maintain health (clearance of invaders) and at the same time, must dampen pro-inflammatory responses to maintain alveolar structure to enable gas exchange. Our failure to understand the cellular and molecular events underlying AM development and biology creates a critical barrier in our attempts to develop new treatment strategies that target the lung. AMs, which constitute >95% of the cells recovered by bronchoalveolar lavage in healthy individuals, are thought to play a central role in lung inflammaging due to their unique regulation by molecular factors in the lung environment.

Indeed, our studies and others have demonstrated an AM inflammatory signature in the elderly (Canan et al., 2014; Wong et al., 2017; Lafuse et al., 2019; Duong et al., 2021). Specifically, AMs from old mice express higher mRNA levels of the macrophage migration inhibitory factor (MIF), TNF-α, IFN-β, IL-10, IL-12p40, and CCL2 than young mice, and old mice also contain higher levels of MIF, IL-1β, IFN-β, and CCL2 in their alveolar lining fluid. Further, old mice have two distinct AM subpopulations, a major CD11c^+^ CD11b^−^ population and a minor CD11c^+^ CD11b^+^ population; the latter is increased significantly in old mice (∼4-fold). Expression of pattern recognition receptors, antigen presentation molecules, and activation markers such as CD206, TLR2, CD16/CD32, MHC class II, and CD86 is higher in CD11c^+^ CD11b^+^ AMs, and these cells express monocytic markers such as Ly6C, CX3CR1, and CD115, suggesting monocytic origin. Old mouse purified CD11c^+^ CD11b^+^ AMs phagocytose significantly more *M. tuberculosis*, and express higher RNA levels of genes favoring *M. tuberculosis* intracellular growth. Thus, the lungs of old mice contain two distinct AM subpopulations, a defined resident subpopulation and an immigrating CD11c^+^ CD11b^+^ AM subpopulation expressing monocytic markers, a unique inflammatory signature, with enhanced *M. tuberculosis* phagocytosis capacity and *M. tuberculosis* survival. Our findings suggest that this CD11c^+^ CD11b^+^ AM subpopulation could be targeted by respiratory pathogens as a niche for replication and survival within the lung of the elderly population driving their susceptibility to respiratory infections.

Nuclear receptors (NRs) function as cellular transcription factors enabling macrophages and other myeloid cells to sense their local environment and shape their immune and metabolic responses (Murphy and Crean, 2015; Leopold Wager et al., 2019a; Bichiou et al., 2021). Drugs targeting NRs represent 16% of all drugs approved for sale in the United States in 2019, demonstrating their feasibility as host-directed therapies (HDTs) for disease management (Ricote and Glass, 2007; Santos et al., 2017; Boudreaux et al., 2019; Zhang et al., 2020). NRs are increasingly appreciated for their importance during aging (Speeckaert et al., 2014; Paillasse and de Medina, 2015; Cisneros and García-Aguirre, 2020; Xu et al., 2020). Most past studies have focused on specific NRs in cell lines or murine cells, thus their function and interactions within primary cells and the lung environment are largely unexplored. Studies using human and murine macrophages (Zollner and Trauner, 2009; Dhiman et al., 2018; Leopold Wager et al., 2019a; Leopold Wager et al., 2019b; Ning et al., 2019; Zhao et al., 2019; Zhao et al., 2020) in cancer (Zhou et al., 2014; Dhiman et al., 2018; Zhao et al., 2019), auto-immunity (Zollner and Trauner, 2009; Lee et al., 2011; Ning et al., 2019; Olson et al., 2020) and infectious diseases (Renga et al., 2012; Leopold Wager et al., 2019a; Leopold Wager et al., 2019b) have highlighted the importance of NRs in maintaining tissue homeostasis (Nagy et al., 2013; Zhao et al., 2020; Alatshan and Benkő, 2021). Several NRs are postulated to dampen pro-inflammatory networks (De Bosscher et al., 2006; Huang and Glass, 2010; Mandard and Patsouris, 2013; Li et al., 2015; Wang et al., 2017; Duez and Pourcet, 2021; Pulakazhi Venu et al., 2021; Vuttaradhi et al., 2021). Thus, NR dysfunction during aging may remove the brakes to pro-inflammatory and oxidative stress pathways with associated altered mitochondrial function as discussed above. Since NRs regulate metabolism and inflammation in a tissue-specific manner, studying NR expression and activity as well as their involvement in altering lung macrophage lipid metabolism, cellular energy homeostasis and protective immune responses during aging could help define new biological pathways that play important roles in host responses in the lung as we age.

One particularly important NR regulating AM development and function is peroxisome proliferator-activated receptor gamma (PPARγ), where our recent work has identified novel targetable PPARγ effectors that regulate inflammation and cell death pathways (Arnett et al., 2018). PPARγ endogenous ligands include eicosanoids, which are critical determinants of cellular metabolism and immune responses that exert both anti- and pro-inflammatory actions (Nagy et al., 2012; Dennis and Norris, 2015; Arnett et al., 2018; Bougarne et al., 2018), and likely play important roles in controlling cellular aging. Indeed, PPARs regulate gene expression through multiple mechanisms, including heterodimerizing with the retinoid X receptor (RXR), and binding to PPAR response elements in promoters. PPARs also trans-repress NF-κB and other transcription factors to negatively regulate pro-inflammatory pathways and stabilize the NR co-repressor NCoR, to repress gene expression (Ricote and Glass, 2007). Using macrophage-specific PPARγ knockout mice (Odegaard et al., 2007), we showed that PPARγ specifically in lung macrophages is important for controlling bacterial growth (e.g. *M. tuberculosis*) and limiting inflammation *in vivo* (Guirado et al., 2018). These findings highlight a critical role of PPARγ in controlling inflammation, yet the impact of aging on PPARγ and expression and activity of other NRs is still unclear.

Based on our knowledge in the field (Oren et al., 1963; O'Neill et al., 2016; Viola et al., 2019), several NRs are postulated to dampen pro-inflammatory networks (De Bosscher et al., 2006; Huang and Glass, 2010; Mandard and Patsouris, 2013; Papi et al., 2014; Leopold Wager et al., 2019a; Klepsch et al., 2019; Duez and Pourcet, 2021). Thus, it is plausible that NR expression and activity are altered across the continuum of age and NRs become dysfunctional; where age-related NR changes can induce aberrant lung macrophage inflammation, oxidative stress, lipid metabolism, and energy programs. Thus, modulation of NR activity with agonists or antagonists could restore healthy immune homeostasis to slow or reverse the lung health issues associated with aging, allowing for more robust protection against lung infections and inflammatory diseases.

### Other Cellular Changes in the Alveolar Compartment During Aging

As we age, alveolar compartment cells become senescent due to the shortening of telomeres and the accumulation of DNA lesions driven by free oxidative radicals generated in the lung environment. AMs, interstitial macrophages, neutrophils and dendritic cells lose their microbicidal and antigen presenting capacities (Wenisch et al., 2000; Uyemura et al., 2002; Stout et al., 2005). Notably, neutrophils exhibit an alteration of their homeostatic functions that may contribute to the susceptibility to lung infections of the elderly (Barkaway et al., 2021). These include decreased phagocytosis, reduced production of ROS and extracellular traps (NETS) formation (Hazeldine et al., 2014), as well as reduced apoptosis and chemotaxis accompanied with dysregulated migration (Niwa et al., 1989; Fulop et al., 2004). Natural killer (NK) cells, which also constitute a first line of defense against virus-infected and malignant cells (Hazeldine and Lord, 2013) change with age. In particular, NK cells become dysfunctional for secretion of regulatory and anti-microbial responses, cytotoxic capacity, and elimination of transformed and senescent cells (Hazeldine and Lord, 2013). These alterations have implications for age-associated immune responses in the alveolar space of elderly individuals. Indeed, several studies implicate NK dysfunction to increased cases of TB reactivation in the elderly, as well as poor resolution of inflammatory disorders and increased incidence of respiratory infections (Hazeldine and Lord, 2013).

With regard to adaptive immunity, the number of B cells decrease during aging leading to reduced immunoglobulin diversity and affinity (Han et al., 2003; McElhaney et al., 2016; Frasca et al., 2020). Age associated inflammation is also known to impact B cell function (Hagen and Derudder, 2020), impacting germinal center formation and cytokine mediated signaling. Systemic T cell proliferation is impaired, repertoire diversity decreases, homeostasis is modified, intracellular signal transduction capability is dysregulated, and T cells produce less cytokines and are less cytotoxic (Fülöp et al., 1999; Voehringer et al., 2002; Ouyang et al., 2003; Naylor et al., 2005). Indeed, CD4 and CD8 T cells show age-related changes in function (Maue et al., 2009; Lorenzo et al., 2018; Haynes, 2020), with changes in CD4 T cells during aging (Haynes and Swain, 2012; Lee et al., 2012) influencing both CD8 T function and B cell antibody production in response to influenza (Maue et al., 2009; Toapanta and Ross, 2009; Lorenzo et al., 2018). For CD4 T cells, intrinsic defects promote delayed adaptive immune responses and recovery from influenza infection, but adoptive transfer studies have also shown that extrinsic factors also contribute to poor T cell function in the lung. This eludes to the influence of the myriad of changes in the alveolar space, such as oxidation of innate molecules or altered AM function, with increasing age that have wide reaching effect including an influence on adaptive immune function. Significant to lung function in older age, memory T cell populations start to lose their function, most notable in tissue specific resident memory CD8 T cells (CD8 T_RM_ cells) (Schenkel et al., 2014; McMaster et al., 2015; Goplen et al., 2020) (Hakim and Gress, 2007). CD8 T_RM_ cells from old mice have been shown to drive lung fibrosis following influenza, SARS-CoV-2 and chikungunya infections, potentially *via* TGFβ, likely delaying recovering of lung function and promoting conditions for secondary infection and other age associated lung disorders (Uhrlaub et al., 2016; Goplen et al., 2020; Goplen et al., 2021; Shenoy and Mizgerd, 2021). Interestingly, within the lung environment, we have also shown that resident effector CD8^+^ T cells demonstrate a robust innate-like response, driven by an IL-12 dependent but antigen-independent secretion of IFNγ (Vesosky et al., 2006a; Vesosky et al., 2006b; Rottinghaus et al., 2009; Vesosky et al., 2009). Such CD8 effector cells were shown to play a role in early innate control of *M. tuberculosis* infection in old mice (Vesosky et al., 2006a; Rottinghaus et al., 2009; Vesosky et al., 2009). The relevance of such cells is not clear, but it is thought to be a compensatory mechanism to establish an additional defense barrier against respiratory pathogens in the elderly (Piergallini and Turner, 2018b), but could equally be one that interferes with optimal antigen specific immunity or perhaps contributes to lung tissue damage that has long term consequences on pulmonary function with increasing age. The study of adaptive immune cells specifically in the lung during aging, in the context of chronic disease such as chronic obstructive pulmonary disease or asthma, is an as yet understudied area (Lee et al., 2012) but lung tissue specificity and age-associated changes likely also play a contributing role to the ability of T and B cells to exert their immune control as we age.

## Discussion

Aging is accompanied by a decline in immune efficacy resulting in increased vulnerability to infections. There is increasing evidence that immune dysfunction linked to exacerbated inflammatory reactivity is associated with failure in organ systems (ex: lung, brain, skeletal muscle) leading to functional decline, morbidity and mortality. Several of these syndromes and diseases such as frailty, dementia, cardiovascular disease and cancer, result in elderly persons being dependent on others for daily living. Immune decline occurs at different ages, and can be influenced by host genetics as well as external “perturbations” such as metabolic status (e.g., dysglycemia) and lifetime exposure to pathogens. The mechanisms underlying these alterations are poorly understood; where it is unclear which changes are primary consequences of aging, and which are compensatory. Furthermore, it is unclear if inflammaging begins in the periphery and spills over into organs such as the lung or *vice versa*. Answering these questions is critical for the development and implementation of therapeutics that delay, prevent or restore an individual’s age-related decrease in immunity, including its associated failure of organ systems. Elucidation of these interactions is best accomplished through longitudinal studies spanning decades, which are logistically challenging and costly. Alternatives are cross-sectional studies in individuals of all ages, or short longitudinal assessments of individuals challenged by an “insult” to their immune system that accelerates aging of their immune cells. These studies in humans present interpretation challenges due to high levels of variability in factors of interest that may underlie responsivity to challenges, for example patients with undiagnosed dysglycemia which is the most prevalent metabolic perturbation in the elderly (Chia et al., 2018; Scordo et al., 2021).

We postulate that there is a gradual increase in inflammation and altered metabolism across the aging continuum, potentially initiated in the pulmonary space, which is directly exposed to environmental insults, that culminates in a cascade of organ and systemic dysfunctions, reaching a peak or *tipping point(s)* where inflammation and oxidation can no longer be reversed by interventions (Figure 2). Modulating cellular oxidative stress-related factors that occur during inflammaging in the alveolus prior to the *tipping point(s)* may allow for the restoration and maintenance of healthy immune and metabolic homeostasis during aging.

## References

[B1] Alatshan A. Benkő S. (2021). Nuclear Receptors as Multiple Regulators of NLRP3 Inflammasome Function. Front. Immunol. 12, 630569. 10.3389/fimmu.2021.630569 33717162 PMC7952630

[B2] Allard B. Panariti A. Martin J. G. (2018). Alveolar Macrophages in the Resolution of Inflammation, Tissue Repair, and Tolerance to Infection. Front. Immunol. 9, 1777. 10.3389/fimmu.2018.01777 30108592 PMC6079255

[B3] Anderson A. J. Jackson T. D. Stroud D. A. Stojanovski D. (2019). Mitochondria-hubs for Regulating Cellular Biochemistry: Emerging Concepts and Networks. Open Biol. 9 (8), 190126. 10.1098/rsob.190126 31387448 PMC6731593

[B4] Arcos J. Diangelo L. E. Scordo J. M. Sasindran S. J. Moliva J. I. Turner J. (2015). Lung Mucosa Lining Fluid Modification ofMycobacterium Tuberculosisto Reprogram Human Neutrophil Killing Mechanisms. J. Infect. Dis. 212 (6), 948–958. 10.1093/infdis/jiv146 25748325 PMC4548464

[B5] Arcos J. Sasindran S. J. Fujiwara N. Turner J. Schlesinger L. S. Torrelles J. B. (2011). Human Lung Hydrolases DelineateMycobacterium Tuberculosis-Macrophage Interactions and the Capacity to Control Infection. J.I. 187 (1), 372–381. 10.4049/jimmunol.1100823 PMC4201034 21602490

[B6] Arcos J. Sasindran S. J. Moliva J. I. Scordo J. M. Sidiki S. Guo H. (2017). *Mycobacterium tuberculosis* Cell wall Released Fragments by the Action of the Human Lung Mucosa Modulate Macrophages to Control Infection in an IL-10-dependent Manner. Mucosal Immunol. 10 (5), 1248–1258. 10.1038/mi.2016.115 28000679 PMC5479761

[B7] Arnett E. Weaver A. M. Woodyard K. C. Montoya M. J. Li M. Hoang K. V. (2018). PPARγ Is Critical for *Mycobacterium tuberculosis* Induction of Mcl-1 and Limitation of Human Macrophage Apoptosis. Plos Pathog. 14 (6), e1007100. 10.1371/journal.ppat.1007100 29928066 PMC6013021

[B8] Barkaway A. Rolas L. Joulia R. Bodkin J. Lenn T. Owen-Woods C. (2021). Age-related Changes in the Local Milieu of Inflamed Tissues Cause Aberrant Neutrophil Trafficking and Subsequent Remote Organ Damage. Immunity 54 (7), 1494–1510. 10.1016/j.immuni.2021.04.025 34033752 PMC8284598

[B9] Bichiou H. Bouabid C. Rabhi I. Guizani-Tabbane L. (2021). Transcription Factors Interplay Orchestrates the Immune-Metabolic Response of Leishmania Infected Macrophages. Front. Cel. Infect. Microbiol. 11, 660415. 10.3389/fcimb.2021.660415 PMC8058464 33898331

[B10] Boudreaux S. P. Duren R. P. Call S. G. Nguyen L. Freire P. R. Narayanan P. (2019). Drug Targeting of NR4A Nuclear Receptors for Treatment of Acute Myeloid Leukemia. Leukemia 33 (1), 52–63. 10.1038/s41375-018-0174-1 29884904 PMC6286710

[B11] Bougarne N. Weyers B. Desmet S. J. Deckers J. Ray D. W. Staels B. (2018). Molecular Actions of PPARα in Lipid Metabolism and Inflammation. Endocr. Rev. 39 (5), 760–802. 10.1210/er.2018-00064 30020428

[B12] Bulut O. Kilic G. Domínguez-Andrés J. Netea M. G. (2020). Overcoming Immune Dysfunction in the Elderly: Trained Immunity as a Novel Approach. Int. Immunol. 32 (12), 741–753. 10.1093/intimm/dxaa052 32766848 PMC7680842

[B13] Canan C. H. Gokhale N. S. Carruthers B. Lafuse W. P. Schlesinger L. S. Torrelles J. B. (2014). Characterization of Lung Inflammation and its Impact on Macrophage Function in Aging. J. Leukoc. Biol. 96 (3), 473–480. 10.1189/jlb.4a0214-093rr 24935957 PMC4632167

[B14] Chalise H. N. (2019). Aging: Basic Concept. Am. J. Biomed. Sci. Res. 1 (1), 8–10. 10.34297/ajbsr.2019.01.000503

[B15] Chia C. W. Egan J. M. Ferrucci L. (2018). Age-Related Changes in Glucose Metabolism, Hyperglycemia, and Cardiovascular Risk. Circ. Res. 123 (7), 886–904. 10.1161/circresaha.118.312806 30355075 PMC6205735

[B16] Cho S. J. Stout-Delgado H. W. (2020). Aging and Lung Disease. Annu. Rev. Physiol. 82, 433–459. 10.1146/annurev-physiol-021119-034610 31730381 PMC7998901

[B17] Cisneros B. García-Aguirre I. (2020). Nuclear Protein export Pathway in Aging Therapeutics. Aging 12 (6), 4682–4684. 10.18632/aging.102948 32200356 PMC7138584

[B18] Davies L. C. Taylor P. R. (2015). Tissue‐resident Macrophages: Then and Now. Immunology 144 (4), 541–548. 10.1111/imm.12451 25684236 PMC4368161

[B19] De Bosscher K. Vanden Berghe W. Haegeman G. (2006). Cross-talk between Nuclear Receptors and Nuclear Factor κB. Oncogene 25 (51), 6868–6886. 10.1038/sj.onc.1209935 17072333

[B20] Dennis E. A. Norris P. C. (2015). Eicosanoid Storm in Infection and Inflammation. Nat. Rev. Immunol. 15 (8), 511–523. 10.1038/nri3859 26139350 PMC4606863

[B21] Dhiman V. K. Bolt M. J. White K. P. (2018). Nuclear Receptors in Cancer - Uncovering New and Evolving Roles through Genomic Analysis. Nat. Rev. Genet. 19 (3), 160–174. 10.1038/nrg.2017.102 29279606

[B22] Duez H. Pourcet B. (2021). Nuclear Receptors in the Control of the NLRP3 Inflammasome Pathway. Front. Endocrinol. 12, 630536. 10.3389/fendo.2021.630536 PMC7947301 33716981

[B23] Duong L. Radley H. Lee B. Dye D. Pixley F. Grounds M. (2021). Macrophage Function in the Elderly and Impact on Injury Repair and Cancer. Immun. Ageing 18 (1), 4. 10.1186/s12979-021-00215-2 33441138 PMC7805172

[B24] Dyer C. (2012). The Interaction of Ageing and Lung Disease. Chron. Respir. Dis. 9 (1), 63–67. 10.1177/1479972311433766 22308556

[B25] Franceschi C. Bonafè M. Valensin S. Olivieri F. De Luca M. Ottaviani E. (2000). Inflamm-aging. An Evolutionary Perspective on Immunosenescence. Ann. N. Y Acad. Sci. 908, 244–254. 10.1111/j.1749-6632.2000.tb06651.x 10911963

[B26] Frasca D. Blomberg B. B. Garcia D. Keilich S. R. Haynes L. (2020). Age‐related Factors that Affect B Cell Responses to Vaccination in Mice and Humans. Immunol. Rev. 296 (1), 142–154. 10.1111/imr.12864 32484934 PMC7371527

[B27] Frasca D. Blomberg B. B. (2016). Inflammaging Decreases Adaptive and Innate Immune Responses in Mice and Humans. Biogerontology 17 (1), 7–19. 10.1007/s10522-015-9578-8 25921609 PMC4626429

[B28] Fronius M. Clauss W. G. Althaus M. (2012). Why Do We Have to Move Fluid to Be Able to Breathe? Front. Physio. 3, 146. 10.3389/fphys.2012.00146 PMC3357553 22661953

[B29] Fulop T. Larbi A. Dupuis G. Le Page A. Frost E. H. Cohen A. A. (2017). Immunosenescence and Inflamm-Aging as Two Sides of the Same Coin: Friends or Foes? Front. Immunol. 8, 1960. 10.3389/fimmu.2017.01960 29375577 PMC5767595

[B30] Fülöp T. Jr. Gagné D. Goulet A.-C. Desgeorges S. Lacombe G. Arcand M. (1999). Age-related Impairment of P56lck and ZAP-70 Activities in Human T Lymphocytes Activated through the TcR/CD3 Complex. Exp. Gerontol. 34 (2), 197–216. 10.1016/s0531-5565(98)00061-8 10363787

[B31] Fulop T. Larbi A. Douziech N. Fortin C. Guérard K.-P. Lesur O. (2004). Signal Transduction and Functional Changes in Neutrophils with Aging. Aging Cell 3 (4), 217–226. 10.1111/j.1474-9728.2004.00110.x 15268755

[B32] Gómez-Serrano M. Camafeita E. Loureiro M. Peral B. (2018). Mitoproteomics: Tackling Mitochondrial Dysfunction in Human Disease. Oxid Med. Cel Longev 2018, 1435934. 10.1155/2018/1435934 PMC6250043 30533169

[B33] Goplen N. P. Wu Y. Son Y. M. Li C. Wang Z. Cheon I. S. (2020). Tissue-resident CD8+ T Cells Drive Age-Associated Chronic Lung Sequelae after Viral Pneumonia. Sci. Immunol. 5 (53). 10.1126/sciimmunol.abc4557 PMC7970412 33158975

[B34] Goplen N. P. Cheon I. S. Sun J. (2021). Age-Related Dynamics of Lung-Resident Memory CD8+ T Cells in the Age of COVID-19. Front. Immunol. 12, 636118. 10.3389/fimmu.2021.636118 33854506 PMC8039372

[B35] Guirado E. Rajaram M. V. Chawla A. Daigle J. La Perle K. M. Arnett E. (2018). Deletion of PPARγ in Lung Macrophages Provides an Immunoprotective Response against *M. tuberculosis* Infection in Mice. Tuberculosis 111, 170–177. 10.1016/j.tube.2018.06.012 30029904 PMC6481684

[B36] Guth A. M. Janssen W. J. Bosio C. M. Crouch E. C. Henson P. M. Dow S. W. (2009). Lung Environment Determines Unique Phenotype of Alveolar Macrophages. Am. J. Physiology-Lung Cell Mol. Physiol. 296 (6), L936–L946. 10.1152/ajplung.90625.2008 PMC2692811 19304907

[B37] Haas R. H. (2019). Mitochondrial Dysfunction in Aging and Diseases of Aging. Biology (Basel) 8 (2). 10.3390/biology8020048 PMC6627182 31213034

[B38] Hagen M. Derudder E. (2020). Inflammation and the Alteration of B-Cell Physiology in Aging. Gerontology 66 (2), 105–113. 10.1159/000501963 31553969

[B39] Hakim F. T. Gress R. E. (2007). Immunosenescence: Deficits in Adaptive Immunity in the Elderly. Tissue Antigens 70 (3), 179–189. 10.1111/j.1399-0039.2007.00891.x 17661905

[B40] Han S. Yang K. Ozen Z. Peng W. Marinova E. Kelsoe G. (2003). Enhanced Differentiation of Splenic Plasma Cells but Diminished Long-Lived High-Affinity Bone Marrow Plasma Cells in Aged Mice. J. Immunol. 170 (3), 1267–1273. 10.4049/jimmunol.170.3.1267 12538685

[B41] Harpur C. M. Le Page M. A. Tate M. D. (2021). Too young to die? How aging affects cellular innate immune responses to influenza virus and disease severity. Virulence 12 (1), 1629–1646. 10.1080/21505594.2021.1939608 34152253 PMC8218692

[B42] Haynes L. (2020). Aging of the Immune System: Research Challenges to Enhance the Health Span of Older Adults. Front. Aging 1, 1–2. 10.3389/fragi.2020.602108 PMC9261332 35822168

[B43] Haynes L. Swain S. L. (2012). Aged-related Shifts in T Cell Homeostasis lead to Intrinsic T Cell Defects. Semin. Immunol. 24 (5), 350–355. 10.1016/j.smim.2012.04.001 22564707 PMC3415577

[B44] Hazeldine J. Harris P. Chapple I. L. Grant M. Greenwood H. Livesey A. (2014). Impaired Neutrophil Extracellular Trap Formation: a Novel Defect in the Innate Immune System of Aged Individuals. Aging Cell 13 (4), 690–698. 10.1111/acel.12222 24779584 PMC4326942

[B45] Hazeldine J. Lord J. M. (2013). The Impact of Ageing on Natural Killer Cell Function and Potential Consequences for Health in Older Adults. Ageing Res. Rev. 12 (4), 1069–1078. 10.1016/j.arr.2013.04.003 23660515 PMC4147963

[B46] Hill P. J. Scordo J. M. Arcos J. Kirkby S. E. Wewers M. D. Wozniak D. J. (2017). Modifications of *Pseudomonas aeruginosa* Cell Envelope in the Cystic Fibrosis Airway Alters Interactions with Immune Cells. Sci. Rep. 7 (1), 4761. 10.1038/s41598-017-05253-9 28684799 PMC5500645

[B47] Hoeksema M. A. Glass C. K. (2019). Nature and Nurture of Tissue-specific Macrophage Phenotypes. Atherosclerosis 281, 159–167. 10.1016/j.atherosclerosis.2018.10.005 30343819 PMC6399046

[B48] Huang W. Glass C. K. (2010). Nuclear Receptors and Inflammation Control: Molecular Mechanisms and Pathophysiological Relevance. Atvb 30 (8), 1542–1549. 10.1161/atvbaha.109.191189 PMC2911147 20631355

[B49] Iddir M. Brito A. Dingeo G. Fernandez Del Campo S. S. Samouda H. La Frano M. R. (2020). Strengthening the Immune System and Reducing Inflammation and Oxidative Stress through Diet and Nutrition: Considerations during the COVID-19 Crisis. Nutrients 12 (6). 10.3390/nu12061562 PMC7352291 32471251

[B50] Ingram T. Chakrabarti L. (2016). Proteomic Profiling of Mitochondria: what Does it Tell Us about the Ageing Brain? Aging 8 (12), 3161–3179. 10.18632/aging.101131 27992860 PMC5270661

[B51] Janssens J. P. Pache J. C. Nicod L. P. (1999). Physiological Changes in Respiratory Function Associated with Ageing. Eur. Respir. J. 13 (1), 197–205. 10.1034/j.1399-3003.1999.13a36.x 10836348

[B52] Jones D. P. Mody V. C. Jr. Carlson J. L. Lynn M. J. Sternberg P. Jr. (2002). Redox Analysis of Human Plasma Allows Separation of Pro-oxidant Events of Aging from Decline in Antioxidant Defenses. Free Radic. Biol. Med. 33 (9), 1290–1300. 10.1016/s0891-5849(02)01040-7 12398937

[B53] Keilich S. R. Bartley J. M. Haynes L. (2019). Diminished Immune Responses with Aging Predispose Older Adults to Common and Uncommon Influenza Complications. Cell Immunol. 345, 103992. 10.1016/j.cellimm.2019.103992 31627841 PMC6939636

[B54] Kim S.-J. Cheresh P. Jablonski R. Williams D. Kamp D. (2015). The Role of Mitochondrial DNA in Mediating Alveolar Epithelial Cell Apoptosis and Pulmonary Fibrosis. Ijms 16 (9), 21486–21519. 10.3390/ijms160921486 26370974 PMC4613264

[B55] Klepsch V. Moschen A. R. Tilg H. Baier G. Hermann-Kleiter N. (2019). Nuclear Receptors Regulate Intestinal Inflammation in the Context of IBD. Front. Immunol. 10, 1070. 10.3389/fimmu.2019.01070 31139192 PMC6527601

[B56] Lafuse W. P. Rajaram M. V. S. Wu Q. Moliva J. I. Torrelles J. B. Turner J. (2019). Identification of an Increased Alveolar Macrophage Subpopulation in Old Mice that Displays Unique Inflammatory Characteristics and Is Permissive toMycobacterium tuberculosisInfection. J.I. 203 (8), 2252–2264. 10.4049/jimmunol.1900495 PMC6783358 31511357

[B57] Lee J. M. Lee Y. K. Mamrosh J. L. Busby S. A. Griffin P. R. Pathak M. C. (2011). A Nuclear-receptor-dependent Phosphatidylcholine Pathway with Antidiabetic Effects. Nature 474 (7352), 506–510. 10.1038/nature10111 21614002 PMC3150801

[B58] Lee N. Shin M. S. Kang I. (2012). T-cell Biology in Aging, with a Focus on Lung Disease. Journals Gerontol. Ser. A: Biol. Sci. Med. Sci. 67A (3), 254–263. 10.1093/gerona/glr237 PMC3297764 22396471

[B59] Leopold Wager C. M. Arnett E. Schlesinger L. S. (2019). Macrophage Nuclear Receptors: Emerging Key Players in Infectious Diseases. Plos Pathog. 15 (3), e1007585–e. 10.1371/journal.ppat.1007585 30897154 PMC6428245

[B60] Leopold Wager C. M. Arnett E. Schlesinger L. S. (2019). *Mycobacterium tuberculosis* and Macrophage Nuclear Receptors: what We Do and Don't Know. Tuberculosis 116, S98–S106. 10.1016/j.tube.2019.04.016 PMC6750772 31060958

[B61] Li L. Liu Y. Chen H.-z. Li F.-w. Wu J.-f. Zhang H.-k. (2015). Impeding the Interaction between Nur77 and P38 Reduces LPS-Induced Inflammation. Nat. Chem. Biol. 11 (5), 339–346. 10.1038/nchembio.1788 25822914

[B62] Linton P. J. Dorshkind K. (2004). Age-related Changes in Lymphocyte Development and Function. Nat. Immunol. 5 (2), 133–139. 10.1038/ni1033 14749784

[B63] Liu G. Summer R. (2019). Cellular Metabolism in Lung Health and Disease. Annu. Rev. Physiol. 81, 403–428. 10.1146/annurev-physiol-020518-114640 30485759 PMC6853603

[B64] López-Otín C. Blasco M. A. Partridge L. Serrano M. Kroemer G. (2013). The Hallmarks of Aging. Cell 153 (6), 1194–1217. 10.1016/j.cell.2013.05.039 23746838 PMC3836174

[B65] Lorenzo E. C. Bartley J. M. Haynes L. (2018). The Impact of Aging on CD4+ T Cell Responses to Influenza Infection. Biogerontology 19 (6), 437–446. 10.1007/s10522-018-9754-8 29616390 PMC6170716

[B66] Lowery E. M. Brubaker A. L. Kuhlmann E. Kovacs E. J. (2013). The Aging Lung. Clin. Interv. Aging 8, 1489–1496. 10.2147/CIA.S51152 24235821 PMC3825547

[B67] Mandard S. Patsouris D. (2013). Nuclear Control of the Inflammatory Response in Mammals by Peroxisome Proliferator-Activated Receptors. PPAR Res. 2013, 613864. 10.1155/2013/613864 23577023 PMC3614066

[B68] Massudi H. Grant R. Braidy N. Guest J. Farnsworth B. Guillemin G. J. (2012). Age-associated Changes in Oxidative Stress and NAD+ Metabolism in Human Tissue. PLoS One 7 (7), e42357. 10.1371/journal.pone.0042357 22848760 PMC3407129

[B69] Maue A. C. Yager E. J. Swain S. L. Woodland D. L. Blackman M. A. Haynes L. (2009). T-cell Immunosenescence: Lessons Learned from Mouse Models of Aging. Trends Immunol. 30 (7), 301–305. 10.1016/j.it.2009.04.007 19541537 PMC3755270

[B70] McElhaney J. E. Kuchel G. A. Zhou X. Swain S. L. Haynes L. (2016). T-cell Immunity to Influenza in Older Adults: A Pathophysiological Framework for Development of More Effective Vaccines. Front. Immunol. 7, 41. 10.3389/fimmu.2016.00041 26941738 PMC4766518

[B71] McMaster S. R. Wilson J. J. Wang H. Kohlmeier J. E. (2015). Airway-Resident Memory CD8 T Cells Provide Antigen-specific Protection against Respiratory Virus Challenge through Rapid IFN-γ Production. J.I. 195 (1), 203–209. 10.4049/jimmunol.1402975 PMC4475417 26026054

[B72] Michaeloudes C. Bhavsar P. K. Mumby S. Xu B. Hui C. K. M. Chung K. F. (2020). Role of Metabolic Reprogramming in Pulmonary Innate Immunity and its Impact on Lung Diseases. J. Innate Immun. 12 (1), 31–46. 10.1159/000504344 31786568 PMC6959099

[B73] Moliva J. I. Duncan M. A. Olmo-Fontánez A. Akhter A. Arnett E. Scordo J. M. (2019). The Lung Mucosa Environment in the Elderly Increases Host Susceptibility to *Mycobacterium tuberculosis* Infection. J. Infect. Dis. 220 (3), 514–523. 10.1093/infdis/jiz138 30923818 PMC6603975

[B74] Moliva J. I. Rajaram M. V. S. Sidiki S. Sasindran S. J. Guirado E. Pan X. J. (2014). Molecular Composition of the Alveolar Lining Fluid in the Aging Lung. Age 36 (3), 9633. 10.1007/s11357-014-9633-4 24584696 PMC4082594

[B75] Murphy E. Crean D. (2015). Molecular Interactions between NR4A Orphan Nuclear Receptors and NF-Κb Are Required for Appropriate Inflammatory Responses and Immune Cell Homeostasis. Biomolecules 5 (3), 1302–1318. 10.3390/biom5031302 26131976 PMC4598753

[B76] Nagy L. Szanto A. Szatmari I. Széles L. (2012). Nuclear Hormone Receptors Enable Macrophages and Dendritic Cells to Sense Their Lipid Environment and Shape Their Immune Response. Physiol. Rev. 92 (2), 739–789. 10.1152/physrev.00004.2011 22535896

[B77] Nagy Z. S. Czimmerer Z. Szanto A. Nagy L. (2013). Pro-inflammatory Cytokines Negatively Regulate PPARγ Mediated Gene Expression in Both Human and Murine Macrophages via Multiple Mechanisms. Immunobiology 218 (11), 1336–1344. 10.1016/j.imbio.2013.06.011 23870825

[B78] Naylor K. Li G. Vallejo A. N. Lee W.-W. Koetz K. Bryl E. (2005). The Influence of Age on T Cell Generation and TCR Diversity. J. Immunol. 174 (11), 7446–7452. 10.4049/jimmunol.174.11.7446 15905594

[B79] Ning L. Lou X. Zhang F. Xu G. (2019). Nuclear Receptors in the Pathogenesis and Management of Inflammatory Bowel Disease. Mediators Inflamm. 2019, 2624941. 10.1155/2019/2624941 30804707 PMC6360586

[B80] Niwa Y. Kasama T. Miyachi Y. Kanoh T. (1989). Neutrophil Chemotaxis, Phagocytosis and Parameters of Reactive Oxygen Species in Human Aging: Cross-Sectional and Longitudinal Studies. Life Sci. 44 (22), 1655–1664. 10.1016/0024-3205(89)90482-7 2733545

[B81] Notter R. H. (2000). Lung Surfactants: Basic Science and Clinical Applications. New York: Marcel Dekker, 1–444.

[B82] O'Neill L. A. J. Kishton R. J. Rathmell J. (2016). A Guide to Immunometabolism for Immunologists. Nat. Rev. Immunol. 16 (9), 553–565. 10.1038/nri.2016.70 27396447 PMC5001910

[B83] Odegaard J. I. Ricardo-Gonzalez R. R. Goforth M. H. Morel C. R. Subramanian V. Mukundan L. (2007). Macrophage-specific PPARγ Controls Alternative Activation and Improves Insulin Resistance. Nature 447 (7148), 1116–1120. 10.1038/nature05894 17515919 PMC2587297

[B84] Olson W. J. Jakic B. Hermann‐Kleiter N. (2020). Regulation of the Germinal center Response by Nuclear Receptors and Implications for Autoimmune Diseases. FEBS J. 287 (14), 2866–2890. 10.1111/febs.15312 32246891 PMC7497069

[B85] Oren R. Farnham A. E. Saito K. Milofsky E. Karnovsky M. L. (1963). Metabolic Patterns in Three Types of Phagocytizing Cells. J. Cel Biol 17, 487–501. 10.1083/jcb.17.3.487 PMC2106210 13940299

[B86] Ouyang Q. Wagner W. M. Voehringer D. Wikby A. Klatt T. Walter S. (2003). Age-associated Accumulation of CMV-specific CD8+ T Cells Expressing the Inhibitory Killer Cell Lectin-like Receptor G1 (KLRG1). Exp. Gerontol. 38 (8), 911–920. 10.1016/s0531-5565(03)00134-7 12915213

[B87] Paillasse M. R. de Medina P. (2015). The NR4A Nuclear Receptors as Potential Targets for Anti-aging Interventions. Med. Hypotheses 84 (2), 135–140. 10.1016/j.mehy.2014.12.003 25543265

[B88] Papi A. De Carolis S. Bertoni S. Storci G. Sceberras V. Santini D. (2014). PPARγ and RXR Ligands Disrupt the Inflammatory Cross-Talk in the Hypoxic Breast Cancer Stem Cells Niche. J. Cel. Physiol 229 (11), 1595–1606. 10.1002/jcp.24601 24604522

[B89] Papp A. C. Azad A. K. Pietrzak M. Williams A. Handelman S. K. Igo R. P. Jr. (2018). AmpliSeq Transcriptome Analysis of Human Alveolar and Monocyte-Derived Macrophages over Time in Response to *Mycobacterium tuberculosis* Infection. PLoS One 13 (5), e0198221. 10.1371/journal.pone.0198221 29847580 PMC5976201

[B90] Pawelec G. Derhovanessian E. Larbi A. (2010). Immunosenescence and Cancer. Crit. Rev. Oncology/Hematology 75 (2), 165–172. 10.1016/j.critrevonc.2010.06.012 20656212

[B91] Piergallini T. J. Turner J. (2018). Tuberculosis in the Elderly: Why Inflammation Matters. Exp. Gerontol. 105, 32–39. 10.1016/j.exger.2017.12.021 29287772 PMC5967410

[B92] Piergallini T. J. Turner J. (2018). Tuberculosis in the Elderly: Why Inflammation Matters. Exp. Gerontol. 105, 32–39. 10.1016/j.exger.2017.12.021 29287772 PMC5967410

[B93] Publications U. N. (2019). World Population Ageing 2019: Highlights2020. United Nations New York.

[B94] Pulakazhi Venu V. K. Alston L. Iftinca M. Tsai Y.-C. Stephens M. Warriyar K. V. V. (2021). Nr4A1 Modulates Inflammation-Associated Intestinal Fibrosis and Dampens Fibrogenic Signaling in Myofibroblasts. Am. J. Physiology-Gastrointestinal Liver Physiol. 321 (3), G280–G297. 10.1152/ajpgi.00338.2019 34288735

[B95] Renga B. Francisci D. D’Amore C. Schiaroli E. Carino A. Baldelli F. (2012). HIV-1 Infection Is Associated with Changes in Nuclear Receptor Transcriptome, Pro-inflammatory and Lipid Profile of Monocytes. BMC Infect. Dis. 12, 274. 10.1186/1471-2334-12-274 23106848 PMC3528633

[B96] Ricote M. Glass C. (2007). PPARs and Molecular Mechanisms of Transrepression. Biochim. Biophys. Acta (Bba) - Mol. Cel Biol. Lipids 1771 (8), 926–935. 10.1016/j.bbalip.2007.02.013 PMC1986735 17433773

[B97] Rottinghaus E. K. Vesosky B. Turner J. (2009). Interleukin-12 Is Sufficient to Promote Antigen-independent Interferon-Gamma Production by CD8 T Cells in Old Mice. Immunology 128 (1 Suppl. l), e679–90. 10.1111/j.1365-2567.2009.03061.x 19740329 PMC2753923

[B98] Santos R. Ursu O. Gaulton A. Bento A. P. Donadi R. S. Bologa C. G. (2017). A Comprehensive Map of Molecular Drug Targets. Nat. Rev. Drug Discov. 16, 19–34. 10.1038/nrd.2016.230 27910877 PMC6314433

[B99] Schenkel J. M. Fraser K. A. Beura L. K. Pauken K. E. Vezys V. Masopust D. (2014). Resident Memory CD8 T Cells Trigger Protective Innate and Adaptive Immune Responses. Science 346 (6205), 98–101. 10.1126/science.1254536 25170049 PMC4449618

[B100] Schneider J. L. Rowe J. H. Garcia-de-Alba C. Kim C. F. Sharpe A. H. Haigis M. C. (2021). The Aging Lung: Physiology, Disease, and Immunity. Cell 184 (8), 1990–2019. 10.1016/j.cell.2021.03.005 33811810 PMC8052295

[B101] Schöttker B. Brenner H. Jansen E. H. Gardiner J. Peasey A. Kubínová R. (2015). Evidence for the Free Radical/oxidative Stress Theory of Ageing from the CHANCES Consortium: a Meta-Analysis of Individual Participant Data. BMC Med. 13, 300. 10.1186/s12916-015-0537-7 26666526 PMC4678534

[B102] Scordo J. M. Aguillón-Durán G. P. Ayala D. Quirino-Cerrillo A. P. Rodríguez-Reyna E. Mora-Guzmán F. (2021). A Prospective Cross-Sectional Study of Tuberculosis in Elderly Hispanics Reveals that BCG Vaccination at Birth Is Protective whereas Diabetes Is Not a Risk Factor. PLoS One 16 (7), e0255194. 10.1371/journal.pone.0255194 34324578 PMC8321126

[B103] Scordo J. M. Arcos J. Kelley H. V. Diangelo L. Sasindran S. J. Youngmin E. (2017). *Mycobacterium tuberculosis* Cell Wall Fragments Released upon Bacterial Contact with the Human Lung Mucosa Alter the Neutrophil Response to Infection. Front. Immunol. 8, 307. 10.3389/fimmu.2017.00307 28373877 PMC5357626

[B104] Scordo J. M. Olmo-Fontánez A. M. Kelley H. V. Sidiki S. Arcos J. Akhter A. (2019). The Human Lung Mucosa Drives Differential *Mycobacterium tuberculosis* Infection Outcome in the Alveolar Epithelium. Mucosal Immunol. 12, 795–804. 10.1038/s41385-019-0156-2 30846830 PMC6462240

[B105] Sekhar M.D. R. V. (2019). “Oxidation Damage Accumulation Aging Theory (The Novel Role of Glutathione),” in Encyclopedia of Gerontology and Population Aging. Editors Gu, D. Dupre M. E. (Cham: Springer International Publishing), 1–9. 10.1007/978-3-319-69892-2_51-1

[B106] Sharma G. Goodwin J. (2006). Effect of Aging on Respiratory System Physiology and Immunology. Clin. Interventions Aging 1 (3), 253–260. 10.2147/ciia.2006.1.3.253 PMC2695176 18046878

[B107] Shenoy A. T. Mizgerd J. P. (2021). Seedy CD8+ TRM Cells in Aging Lungs Drive Susceptibility to Pneumonia and Sequelae. Cell Mol Immunol 18 (4), 787–789. 10.1038/s41423-020-00629-w 33420355 PMC7791330

[B108] Shivshankar P. Boyd A. R. Le Saux C. J. Yeh I.-T. Orihuela C. J. (2011). Cellular Senescence Increases Expression of Bacterial Ligands in the Lungs and Is Positively Correlated with Increased Susceptibility to Pneumococcal Pneumonia. Aging Cell 10 (5), 798–806. 10.1111/j.1474-9726.2011.00720.x 21615674 PMC3173515

[B109] Short K. R. Bigelow M. L. Kahl J. Singh R. Coenen-Schimke J. Raghavakaimal S. (2005). Decline in Skeletal Muscle Mitochondrial Function with Aging in Humans. Proc. Natl. Acad. Sci. 102 (15), 5618–5623. 10.1073/pnas.0501559102 15800038 PMC556267

[B110] Speeckaert M. M. Vanfraechem C. Speeckaert R. Delanghe J. R. (2014). Peroxisome Proliferator-Activated Receptor Agonists in a Battle against the Aging Kidney. Ageing Res. Rev. 14, 1–18. 10.1016/j.arr.2014.01.006 24503003

[B111] Stauch K. L. Purnell P. R. Villeneuve L. M. Fox H. S. (2015). Proteomic Analysis and Functional Characterization of Mouse Brain Mitochondria during Aging Reveal Alterations in Energy Metabolism. Proteomics 15 (9), 1574–1586. 10.1002/pmic.201400277 25546256 PMC4465935

[B112] Stout R. D. Jiang C. Matta B. Tietzel I. Watkins S. K. Suttles J. (2005). Macrophages Sequentially Change Their Functional Phenotype in Response to Changes in Microenvironmental Influences. J. Immunol. 175 (1), 342–349. 10.4049/jimmunol.175.1.342 15972667

[B113] Thomas E. T. Guppy M. Straus S. E. Bell K. J. L. Glasziou P. (2019). Rate of normal Lung Function Decline in Ageing Adults: a Systematic Review of Prospective Cohort Studies. BMJ Open 9 (6), e028150. 10.1136/bmjopen-2018-028150 PMC6597635 31248928

[B114] Toapanta F. R. Ross T. M. (2009). Impaired Immune Responses in the Lungs of Aged Mice Following Influenza Infection. Respir. Res. 10, 112. 10.1186/1465-9921-10-112 19922665 PMC2785782

[B115] Torrelles J. B. Schlesinger L. S. (2017). Integrating Lung Physiology, Immunology, and Tuberculosis. Trends Microbiol. 25 (8), 688–697. 10.1016/j.tim.2017.03.007 28366292 PMC5522344

[B116] Ubaida-Mohien C. Lyashkov A. Gonzalez-Freire M. Tharakan R. Shardell M. Moaddel R. (2019). Discovery Proteomics in Aging Human Skeletal Muscle Finds Change in Spliceosome, Immunity, Proteostasis and Mitochondria. Elife 8, eLife.49874. 10.7554/eLife.49874 PMC6810669 31642809

[B117] Uhrlaub J. L. Pulko V. DeFilippis V. R. Broeckel R. Streblow D. N. Coleman G. D. (2016). Dysregulated TGF-β Production Underlies the Age-Related Vulnerability to Chikungunya Virus. Plos Pathog. 12 (10), e1005891. 10.1371/journal.ppat.1005891 27736984 PMC5063327

[B118] Uyemura K. Castle S. C. Makinodan T. (2002). The Frail Elderly: Role of Dendritic Cells in the Susceptibility of Infection. Mech. Ageing Develop. 123 (8), 955–962. 10.1016/s0047-6374(02)00033-7 12044944

[B119] van Oostrom S. H. Engelfriet P. M. Verschuren W. M. M. Schipper M. Wouters I. M. Boezen M. (2018). Aging-related Trajectories of Lung Function in the General Population-The Doetinchem Cohort Study. PLoS One 13 (5), e0197250. 10.1371/journal.pone.0197250 29768509 PMC5955530

[B120] Vaz Fragoso C. A. Lee P. J. (2012). The Aging Lung. Journals Gerontol. Ser. A: Biol. Sci. Med. Sci. 67A (3), 233–235. 10.1093/gerona/glr249 PMC3297766 22396469

[B121] Vesosky B. Flaherty D. K. Rottinghaus E. K. Beamer G. L. Turner J. (2006). Age Dependent Increase in Early Resistance of Mice to *Mycobacterium tuberculosis* Is Associated with an Increase in CD8 T Cells that Are Capable of Antigen Independent IFN-γ Production. Exp. Gerontol. 41 (11), 1185–1194. 10.1016/j.exger.2006.08.006 17029663

[B122] Vesosky B. Flaherty D. K. Turner J. (2006). Th1 Cytokines Facilitate CD8-T-Cell-Mediated Early Resistance to Infection with *Mycobacterium tuberculosis* in Old Mice. Infect. Immun. 74 (6), 3314–3324. 10.1128/iai.01475-05 16714559 PMC1479270

[B123] Vesosky B. Rottinghaus E. K. Davis C. Turner J. (2009). CD8 T Cells in Old Mice Contribute to the Innate Immune Response to *Mycobacterium tuberculosis* via Interleukin-12p70-dependent and Antigen-independent Production of Gamma Interferon. Infect. Immun. 77 (8), 3355–3363. 10.1128/iai.00295-09 19470747 PMC2715662

[B124] Viola A. Munari F. Sánchez-Rodríguez R. Scolaro T. Castegna A. (2019). The Metabolic Signature of Macrophage Responses. Front. Immunol. 10, 1462. 10.3389/fimmu.2019.01462 31333642 PMC6618143

[B125] Voehringer D. Koschella M. Pircher H. (2002). Lack of Proliferative Capacity of Human Effector and Memory T Cells Expressing Killer Cell Lectinlike Receptor G1 (KLRG1). Blood 100 (10), 3698–3702. 10.1182/blood-2002-02-0657 12393723

[B126] Vuttaradhi V. K. Ezhil I. Ramani D. Kanumuri R. Raghavan S. Balasubramanian V. (2021). Inflammation-induced PELP1 Expression Promotes Tumorigenesis by Activating GM-CSF Paracrine Secretion in the Tumor Microenvironment. J. Biol. Chem. 298 (1), 101406. 10.1016/j.jbc.2021.101406 34774800 PMC8671644

[B127] Wang L. Nanayakkara G. Yang Q. Tan H. Drummer C. Sun Y. (2017). A Comprehensive Data Mining Study Shows that Most Nuclear Receptors Act as Newly Proposed Homeostasis-Associated Molecular Pattern Receptors. J. Hematol. Oncol. 10 (1), 168. 10.1186/s13045-017-0526-8 29065888 PMC5655880

[B128] Wenisch C. Patruta S. Daxböck F. Krause R. Hörl W. (2000). Effect of Age on Human Neutrophil Function. J. Leukoc. Biol. 67 (1), 40–45. 10.1002/jlb.67.1.40 10647996

[B129] Wong C. K. Smith C. A. Sakamoto K. Kaminski N. Koff J. L. Goldstein D. R. (2017). Aging Impairs Alveolar Macrophage Phagocytosis and Increases Influenza-Induced Mortality in Mice. J.I. 199 (3), 1060–1068. 10.4049/jimmunol.1700397 PMC5557035 28646038

[B130] Xu L. Ma X. Verma N. Perie L. Pendse J. Shamloo S. (2020). PPARγ Agonists Delay Age-Associated Metabolic Disease and Extend Longevity. Aging Cell 19 (11), e13267. 10.1111/acel.13267 33219735 PMC7681041

[B131] Yang L. Cao Y. Zhao J. Fang Y. Liu N. Zhang Y. (2019). Multidimensional Proteomics Identifies Declines in Protein Homeostasis and Mitochondria as Early Signals for Normal Aging and Age-Associated Disease in Drosophila*[S]. Mol. Cell Proteomics 18 (10), 2078–2088. 10.1074/mcp.ra119.001621 31434710 PMC6773560

[B132] Zhang C. Zhang B. Zhang X. Sun G. Sun X. (2020). Targeting Orphan Nuclear Receptors NR4As for Energy Homeostasis and Diabetes. Front. Pharmacol. 11, 587457. 10.3389/fphar.2020.587457 33328994 PMC7728612

[B133] Zhao L. Zhou S. Gustafsson J. Å. (2019). Nuclear Receptors: Recent Drug Discovery for Cancer Therapies. Endocr. Rev. 40 (5), 1207–1249. 10.1210/er.2018-00222 30869771

[B134] Zhao L. Gimple R. C. Yang Z. Wei Y. Gustafsson J. A. Zhou S. (2020). Immunoregulatory Functions of Nuclear Receptors: Mechanisms and Therapeutic Implications. Trends Endocrinol. Metab. 31 (2), 93–106. 10.1016/j.tem.2019.10.002 31706690

[B135] Zhou F. Drabsch Y. Dekker T. J. A. de Vinuesa A. G. Li Y. Hawinkels L. J. A. C. (2014). Nuclear Receptor NR4A1 Promotes Breast Cancer Invasion and Metastasis by Activating TGF-β Signalling. Nat. Commun. 5, 3388. 10.1038/ncomms4388 24584437

[B136] Zollner G. Trauner M. (2009). Nuclear Receptors as Therapeutic Targets in Cholestatic Liver Diseases. Br. J. Pharmacol. 156 (1), 7–27. 10.1111/j.1476-5381.2008.00030.x 19133988 PMC2697779

